# A *Scientist Guerilla Fighter* in the Frontiers of Bioinformatics—In Memory of Bailin Hao

**DOI:** 10.1016/j.gpb.2018.11.001

**Published:** 2018-12-06

**Authors:** Jun Yu

**Affiliations:** 1 CAS Key Laboratory of Genome Sciences and Information, Beijing Institute of Genomics, Chinese Academy of Sciences, Beijing 100101, China; 2 University of Chinese Academy of Sciences, Beijing 100049, China

The well-known physicist and bioinformaticist, Professor, Academician of Chinese Academy of Sciences (the Academy), and the former director of Institute of Theoretical Physics (the Institute; 1990–1994), Mr. Bailin Hao, passed away early this year (March 7, 2018). In the cover of this issue of *Genomics, Proteomics & Bioinformatics* (GPB), the Editorial Board has decided to publish his art-processed portrait in memory of our colleague (Prof. Hao had served on the Editorial Board of GPB during 2003–2015), friend, and one of the scientific giants we had worked closely with. Most importantly, we would like to take this opportunity to pay respect to him and his generation of scientists, who may have left us as biological beings, but their personal images, professional works, and scientific spirits will certainly be with us forever as heroes, heroines, and role models, especially for the younger generations who may or may not have known them personally.

His much-loved wife, Mrs. Shuyu Zhang, in her recently-published book, *Bailin Hao* — *A Scientist Guerilla Fighter* [1], has so nicknamed Prof. Hao, *guerilla fighter*, referring to the fact that Prof. Hao had been shifting research focuses quite a few times in his over-60-year-long scientific career, under the name of the National Needs at different politically- and economically-defined periods from 1950s to 2010s. He indeed had many research activities in various frontiers of scientific fields within the disciplines of physics, mathematics, statistics, and biology, and had published over 180 peer-reviewed papers, dozens of scientific books, and even more book chapters (including foreign-language translations), which are all closely related to his research interests. In fact, the official announcement accompanying his obituary from the Institute and the Academy has listed over ten such fields (http://www.cas.cn/xb/gz/201803/t20180308_4637577.shtml). In addition, he was also a *tireless fighter* in popularizing sciences as he had written many books and book chapters, as well as articles and blogs, which are also counted by the hundreds in total and cover a broad range of scientific fields he had worked in, including the last one — bioinformatics [1]. Prof. Hao published about 50 peer-reviewed papers in this field, which mostly involve the development of algorithms, software tools, and web servers. His best-acknowledged contribution to the field is his genome sequence analysis tools and analyses for prokaryotic genomes, such as CVTree [2], [3], [4], [5], [6], [7] (http://www.itp.ac.cn/~hao/). All his publications are thoroughly recorded in Mrs. Zhang’s book [1], except his latest article published in this issue, which he had been working on until this March [8]. We, who are younger than Prof. Hao’s generation of scientists, especially those of us who were born in the 1960s, 1970s, and after, may not understand fully why Prof. Hao called himself a *guerilla fighter*. The reasons were stated on the back cover of Mrs. Zhang’s book: “*In the historical transition period, when China emerged from feudal backwardness and humiliations by foreign powers and began to march toward modernization, Bailin Hao belonged to the group who persisted in carrying on their work and struggling to do their best from the beginning to the end. On the one hand, they managed to achieve the best solutions possible under the preexisting historical conditions and contemporary societal limitations; on the other hand, their individual intelligence could not be fully applied to scientific endeavors owing to societal and historical reasons. Their experiences should not be repeated in younger generations, but should be known to them.*”

Although his many professional and personal accomplishments have been reviewed and praised by many of his close friends and students, I still feel that it is necessary to tell one of his lesser-known stories, so our younger bioinformaticists get to know his contributions to the field, his moral principles toward science and profession, and his characters that are common among his generation.

I came to know Prof. Hao personally in the summer of 1999 when I was organizing efforts to sequence the rice genome, known as the Chinese Hybrid Rice Genome Project [9], [10], [11]. Meanwhile, six other initiatives had already been underway to sequence the genome of another rice cultivar [12], which were at different stages of success. The competition was extremely fierce. Aside from the only certainty we had, *i.e.*, acquiring enough raw data, the project faced several uncertainties. Who were the task leaders to develop all software tools concerning sequence assembly, gene prediction, gene annotation, and gene expression modeling? Could we deliver all these tools in time? Could our in-house tools be competitive? The project leadership identified a few senior professors from the Academy and universities to collaborate with on these tasks: Prof. Hao, together with his long-term collaborator Prof. Weimou Zheng of the Academy and Prof. Huimin Xie of Soochow University for the gene prediction tool, Prof. Songgang Li of Peking University for the sequence assembler, and Prof. Guoying Li of the Academy for the gene expression modeling. Prof. Hao took his assignment without blinking an eye. The same spirit and action styles as what Mrs. Zhang had emphasized in her book (as I quoted above) were clearly witnessed again at the very moment of our conversation. The image of Kingdom Shu’s senior general Zhong Huang (a well-known figure from the classic Chinese historical novel *Romance of Three Kingdoms*) somehow appeared in my mind at that very moment; it means full confidence.

The task was new for him; there had been a vast amount of unfamiliar literature for him to read and digest. It was challenging for him; there had been half a dozen software packages either better used for simple genomes but producing unsatisfying results for complex genomes or being developed for complex genomes but not yet publically available. However, as a heroic guerilla fighter, Prof. Hao was always determined to fight to the bitter end with full-hearted efforts, and most importantly, he was not only a veteran in computing sciences, knowing what he does the best, but also an experienced leader in his fields, knowing how to organize a raid and how to assign roles to the best fit. Much of the story was more detailed in Mrs. Zhang’s book, again.

And yet, this is not the end of this gene prediction algorithm story. Prof. Hao had delivered his promise in time, indeed. However, this time, the ball was in my court. After detailed comparison of the gene finding results with various other equivalent software packages, we found that his tool is better than all others by significant but small margins [13]. We then faced a tough choice: if we used our own gene finder, it would be hard to avoid detailed comparison to praise merits of our own package, and by default, we needed to demonstrate that others fell short of something in subtlety. As a matter of honesty and morality, Prof. Hao had decided to publish his gene finder in a more specialized journal instead of quoted usage in our *Science*’s cover story [9]. This move manifests his foreseen concern on roles that may be played by Chinese scientists later and in a larger picture of the scientific landscape where respect and friendship are foremost important than short-term benefits for the individuals involved in the efforts. After all, science and its knowledge are to be promoted and shared by all scientists and their homelands, regardless of their political and religious believes. I still remember the moment when the decision was made. I was staring at Prof. Hao, he was so calm, with his familiar smiles, and when I was looking into his eyes, I saw two pools of deep sea water; and it was so deep and clear as if there was no bottom. As being prepared for the worst, I did encounter many obstacles in completing the scientific endeavor; one of them was a deadly refusal to license the most popular sequence assembling package to Chinese scientists in even public institutions.

We should all learn from Prof. Hao and try our best to be courageous, honest, forward-thinking, and open-minded. One of Prof. Hao’s legacies is to inaugurate a national institution for bioinformatics and its applications (http://blog.sciencenet.cn/blog-1248-237322.html), and he had been involved in several rounds of feasibility studies during ten years after his proposal in 1999. The campaign has been continuously carried on by the next two generations of Chinese Bioinformaticists; the hope is that in one of these days into the future years, this highly-desired symbolic organization is decided to be funded. We all are, after all, marching over the shoulders of many scientific giants toward modern societies, where sciences are highly valued and scientists, as well as their contributions to sciences, are deeply and precisely appreciated and celebrated timely.

## Competing interests

The author has declared that there are no competing interests.
